# Promises and challenges of adoptive T-cell therapies for solid tumours

**DOI:** 10.1038/s41416-021-01353-6

**Published:** 2021-03-29

**Authors:** Matteo Morotti, Ashwag Albukhari, Abdulkhaliq Alsaadi, Mara Artibani, James D. Brenton, Stuart M. Curbishley, Tao Dong, Michael L. Dustin, Zhiyuan Hu, Nicholas McGranahan, Martin L. Miller, Laura Santana-Gonzalez, Leonard W. Seymour, Tingyan Shi, Peter Van Loo, Christopher Yau, Helen White, Nina Wietek, David N. Church, David C. Wedge, Ahmed A. Ahmed

**Affiliations:** 1 Ovarian Cancer Cell Laboratory, MRC Weatherall Institute of Molecular Medicine, University of Oxford, Oxford, UK; 2 Biochemistry Department, Faculty of Science, King Abdulaziz University, Jeddah, Saudi Arabia; 3 Functional Genomics of Ovarian Cancer Laboratory, Cancer Research UK Cambridge Institute, University of Cambridge, Cambridge, UK; 4 Advanced Therapies Facility and National Institute for Health Research (NIHR) Biomedical Research Centre, University of Birmingham, Birmingham, UK; 5 Medical Research Council (MRC) Human Immunology Unit, MRC Weatherall Institute of Molecular Medicine, University of Oxford, Oxford, UK; 6 Chinese Academy of Medical Sciences (CAMS) Oxford Institute, University of Oxford, Oxford, UK; 7 Kennedy Institute of Rheumatology, University of Oxford, Oxford, UK; 8 Cancer Genome Evolution Research Group, University College London Cancer Institute, London, UK; 9 Cancer System Biology Group, Cancer Research UK Cambridge Institute, University of Cambridge, Cambridge, UK; 10 Gene Therapy Group, Department of Oncology, University of Oxford, Oxford, UK; 11 Department of Gynecological Oncology, Zhongshan Hospital, Fudan University, Shanghai, China; 12 Cancer Genomics Laboratory, The Francis Crick Institute, London, UK; 13 Division of Informatics, Imaging and Data Sciences, Faculty of Biology Medicine and Health, University of Manchester, Manchester, UK; 14 The Alan Turing Institute, London, UK; 15 Patient Representative, Endometrial Cancer Genomics England Clinical Interpretation Partnership (GeCIP) Domain, London, UK; 16 Wellcome Centre for Human Genetics, University of Oxford, Oxford, UK; 17 Oxford NIHR Biomedical Research Centre, Oxford, UK; 18 Manchester Cancer Research Centre, University of Manchester, Manchester, UK; 19 Nuffield Department of Women’s & Reproductive Health, University of Oxford, Oxford, UK; 20 Present Address: Department of Oncology, Ludwig Institute for Cancer Research Lausanne, Lausanne University Hospital (CHUV) and University of Lausanne (UNIL), Lausanne, Switzerland

**Keywords:** Immunotherapy, Cancer genomics, Cancer immunotherapy, Tumour immunology

## Abstract

Cancer is a leading cause of death worldwide and, despite new targeted therapies and immunotherapies, many patients with advanced-stage- or high-risk cancers still die, owing to metastatic disease. Adoptive T-cell therapy, involving the autologous or allogeneic transplant of tumour-infiltrating lymphocytes or genetically modified T cells expressing novel T-cell receptors or chimeric antigen receptors, has shown promise in the treatment of cancer patients, leading to durable responses and, in some cases, cure. Technological advances in genomics, computational biology, immunology and cell manufacturing have brought the aspiration of individualised therapies for cancer patients closer to reality. This new era of cell-based individualised therapeutics challenges the traditional standards of therapeutic interventions and provides opportunities for a paradigm shift in our approach to cancer therapy. Invited speakers at a 2020 symposium discussed three areas—cancer genomics, cancer immunology and cell-therapy manufacturing—that are essential to the effective translation of T-cell therapies in the treatment of solid malignancies. Key advances have been made in understanding genetic intratumour heterogeneity, and strategies to accurately identify neoantigens, overcome T-cell exhaustion and circumvent tumour immunosuppression after cell-therapy infusion are being developed. Advances are being made in cell-manufacturing approaches that have the potential to establish cell-therapies as credible therapeutic options. T-cell therapies face many challenges but hold great promise for improving clinical outcomes for patients with solid tumours.

## Background

Personalised, or precision, medicine aims to identify the optimal treatment for each patient in order to maximise benefit and minimise toxicity. In the context of cancer treatment, advances in genomics have enabled the identification of germline and somatic genomic alterations that can be matched to therapeutics on an individual patient basis^1^—in some cases, such as the use of imatinib for the treatment of chronic myeloid leukaemia to great effect.^2^ However, most cancer genomes lack driver mutations for which a molecularly targeted agent is available.^3^ The unprecedented results obtained using novel cancer immunotherapies such as immune checkpoint blockade (ICB) have revealed the potential of leveraging the immune system in cancer treatment.^4^ Unfortunately, despite durable responses in a subset of patients, particularly those with melanoma or non-small cell lung cancer (NSCLC),^5–7^ most patients do not respond to the immunotherapies in current use;^8,9^ in fact, it has been estimated that responders to ICB might constitute around only 13% of cancer patients.^10^

Advanced therapy medicinal products (ATMPs) hold promise to transform cancer treatment^11^—cell-based vaccines,^12,13^ engineered T cells^14^ or autologous tumour-infiltrating lymphocytes (TILs), for example, represent highly personalised modes of cancer treatment. However, the application of cell-therapy approaches to large numbers of cancer patients presents major challenges, as good manufacturing practice (GMP) is a complex and expensive process. Moreover, to improve the effectiveness of ATMPs, we need to gain a better and more comprehensive understanding of the interaction between cancer and the immune system. This review will discuss what we believe are the three most important areas for advancing the field of personalised cell immunotherapies: cancer genomics; cancer–immune system interactions; and ATMP manufacturing. We will focus on discussing the promises and challenges of each area for solid cancers and highlight potential factors for improvement, particularly in the field of T-cell therapy (Box 1).

Box 1 Generation of this conference report This Oxford meeting ‘Advanced Personalised Therapeutics in Solid Cancers’ in February 2020 convened experts to discuss our current understanding of challenges in advance medicinal therapeutic products (ATMPs) in the treatment of solid cancer. The emphasis was on discussing challenges in the field of cancer genomics, cancer–immune system interaction and manufacturing of ATMPs. The idea of summarising the outputs from the meeting in a report was proposed and unanimously approved before the meeting. Following the talks, at the end of each topic session, an open discussion with all meeting participants was held to collate ideas and future perspectives. A draft statement was then circulated to all authors for feedback and refinement, leading to agreement with the views expressed in this manuscript.

## ATMPs in the era of personalised medicine

An ATMP can be a gene therapy medicinal product (e.g. Holoclar®, a stem-cell treatment used for limbal stem-cell deficiency in the eye); a somatic cell therapy medicinal product (e.g. TILs for the treatment of tumours); a tissue-engineered product (e.g. anti-CD19 CAR-T therapies); or a combination of any of the above (Table 1). Personalised cell-therapy treatments are a type of ATMP, manufactured specifically for each patient using their own cellular material (e.g. immune cells). They are intended as long-term or permanent therapeutic solutions to acute or chronic human diseases such as cancer. The ability to extract and grow immune cells—in particular, T-cells—in vitro paved the way for the use of adoptive cell transfer (ACT; the transfer of cells to a patient) in cancer immunotherapy.^15^ Broadly, ACT can be carried out using three different T-cell approaches. In the first, TIL-ACT, endogenous TILs are expanded ex vivo from a patient’s tumour before being infused back into the patient. The second approach uses T-cell receptors (TCRs) that have been engineered to recognise specific tumour antigens, although this approach is limited to major histocompatibility complex (MHC)-expressed antigens. Chimeric antigen receptors (CARs) comprise an extracellular antigen recognition domain, a transmembrane domain, and a cytoplasmic signalling domain, and so can recognise a variety of cell-surface antigens, by contrast, independently of MHCs.^16,17^

**Table 1:** Advanced therapeutic medicinal products (ATMPs).

Product category	Definition	Examples
Gene-therapy medicinal product (GTMP)	Contain genes that lead to a therapeutic, prophylactic or diagnostic effect. They work by inserting ‘recombinant’ genes into the body, usually to treat a variety of diseases, including genetic disorders, cancer or long-term diseases.	Plasmid DNA Viral vectors Genetically engineered micro-organisms Human gene-editing technology Patient-derived cellular gene therapy products
Somatic-cell therapy medicinal product (SCTMP)	Contain cells or tissues that have been manipulated to change their biological characteristics or cells or tissues not intended to be used for the same essential/original functions in the body.	Products containing or consisting of animal cells or tissues Other autologous and allogeneic cells therapies Xenogeneic living cells Stem cells and stem cell-derived products
Tissue-engineered product (TEP)	Contain cells or tissues that have been modified so they can be used to repair, regenerate or replace human tissue.	Products containing or consisting of animal cells or tissues Products might also contain additional substances, such as cellular products, biomolecules, biomaterials, chemical substances, scaffolds or matrices Products for cartilage or cardiac defects, among others Stem cells and stem cells-derived products
Combined ATMP	A combination of the above	None as yet

Scientific advances throughout the entire pipeline from biopsy to the manufacturing of cell therapy products have made ACT potentially more accessible to growing numbers of patients. Accordingly, cell therapy trials in cancer are steadily increasing in number worldwide, with the second largest number of active trials (after ICB) in the immuno-oncology space.^18^ However, despite ~90% of cancer incidences globally being caused by solid tumours, only around half of these trials are targeting solid tumours, and they have rarely been extended to non-melanoma cancers, due to a lower immunogenicity of these tumours.^19–23^ Moreover, although clinical trials using cell therapy products directed against cancer neoantigens derived from somatic mutations hold promise, evidence of success is still limited to case reports of patients with particular tumour types such as cervical cancer,^24^ cholangiocarcinoma,^25^ metastatic melanoma,^26–29^ colorectal cancer,^30^ breast cancer^31^ and thymoma.^32^ Further studies are thus needed to advance the potentially curative approach of ACT.

## Cancer genomics

The decreasing costs and technological advances in massively parallel sequencing techniques have enabled the identification of somatic mutations in cancer on a large scale and facilitated the clinical implementation of genomic medicine.^33–35^

### Neoantigens and personalisation of therapy

The cellular immune response to cancer largely depends on T cells that specifically target cancer/testis antigens (CTA) or cancer neoantigens that are derived from somatic genomic alterations that lead to the expression of immunogenic neoepitopes.^36–38^ In this article, we focus on cancer neoantigens, which result from genomic perturbations, exhibit entirely novel amino acid sequences and, importantly, are rarely shared among patients. These mutant peptides that bind to human leukocyte antigen (HLA) class I or HLA class II molecules are capable of generating a robust and durable immune response, and high mutational and predicted neoantigen load are significantly associated with improved progression-free and overall survival in melanoma patients treated with TIL-ACT.^39^ Furthermore, evidence from melanoma patients treated with TILs suggests that the evoked immune response comprises both CD4^+^ and CD8^+^ T cells specific for mutant epitopes,^25,27,40^ with cases of off-target immune response against the wild-type, non-mutated peptide being exceptionally rare.^41,42^

**Fig. 1:**
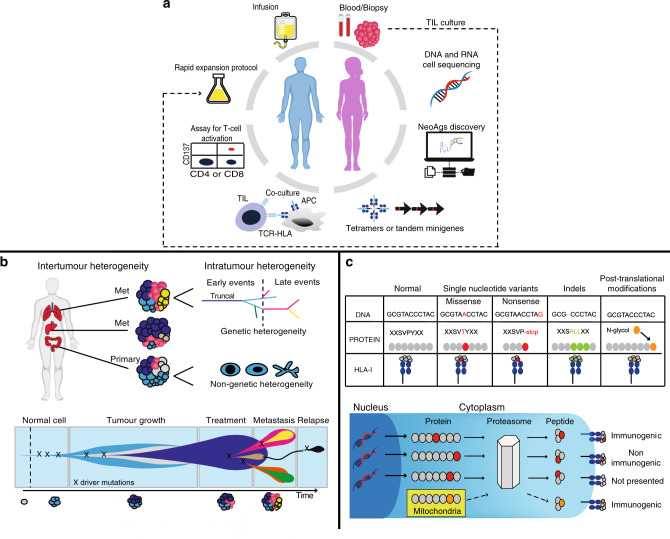
Targeting cancer neoantigens using cell therapy. **a** Using autologous tumour-infiltrating lymphocytes in autologous cell transfer. The resected specimen is divided into multiple tumour fragments that are individually grown in IL-2 for 7–10 days. For the ‘non-specific’ TIL therapy (dashed line) the individual cultures are then moved to a rapid expansion protocol before reinfusion into patients. Neoantigen-TIL therapy involves the sequencing of exomic or whole-genome DNA from tumour cells and healthy cells to identify tumour-specific mutations, before RNA-sequencing is used to check for the expression of mutations. Corresponding minigenes or peptides encoding each mutated amino acid are synthesised and expressed in or pulsed into a patient’s autologous antigen-presenting cells (APCs) for presentation in the context of a patient’s HLA. Individual mutations responsible for tumour recognition are identified by analysing activation of a T-cell co-stimulatory marker, such as 41BB/CD137 (CD8^+^ T cells), in response to cognate target antigen recognition. **b** Genetic and genomic heterogeneity and evolution of clonal populations. Upper panel: Genetic and phenotypic variations are observed between tumours of different tissues (inter-tumour heterogeneity). Within a tumour, subclonal diversity can be observed (intra-tumour heterogeneity, different colours of tumour clones). Clonal alterations occurring early in tumorigenesis are represented by the blue trunk of the phylogenetic tree (truncal mutations); later alterations could be shared by tumour cells in some regions of the tumour (light blue and pink branches of the tree) or present in only one region of the tumour (yellow branches of the tree) in a branched cancer evolution model. Tumour subclones can also show differential gene expression due to non-genetic heterogeneity. Lower row: Unique clones (represented by different colours) emerge as a consequence of accumulating driver mutations in the progeny of a single most recent common ancestor cell. Ongoing linear and branching evolution results in multiple simultaneous subclones that can individually give rise to episodes of disease relapse and metastasis. **c** Overview of the neoantigen landscape. The sources of potential neoantigens for HLA class I ligands are shown. In tumours, mutated or aberrantly expressed proteins are processed via the proteasome into peptides. The cross-priming abilities of peptides are also linked to non-genetic factors such as protein stability, which can be modulated by several factors, including their localisation in the mitochondria. These peptides can be loaded onto HLA class I molecules and might or might not elicit a CD8^+^ T-cell response, depending on several factors, including peptide sequence or T-cell receptor (TCR) sequences. In general, most of the neoantigens derived from single-nucleotide variants gain their immunogenicity through altered amino acids involved in direct T-cell contact.

The combination of genomics and cellular immunotherapy permits the identification of somatic alterations and the prediction of potential neoantigens that could be utilised as targets for therapy by vaccination, adoptive TIL transfer, or engineered T-cells.^42,43^ The pipeline for neoantigen-directed cell-therapy is presented in Fig. 1a. Significant efforts have been made to advance the individual steps, particularly the detection of mutations and prediction of neoepitopes, which is principally determined by the probability that mutant peptides bind HLA-I.^44–47^ The current state-of-the-art and challenges of bioinformatic analysis of the cancer mutanome for ACT has been extensively reviewed elsewhere.^47,48^

### Tumour heterogeneity and evolution

Data from international collaborative studies have shed light on many aspects of cancer genomics and cancer evolution, which could potentially inform the implementation of bioinformatics pipelines for cancer mutanome discovery.^33,49^ Genomic studies have demonstrated how tumour heterogeneity and evolution drive resistance to systemic anti-cancer and targeted therapy^50–52^ such as epidermal growth factor receptor (EGFR) amplification and mutations in the MET or PIK3CA gene resistance in EGFR+ tumours after EGFR inhibitors treatment.^53^ Cancer heterogeneity similarly represents a pivotal challenge for the development of neoantigen-directed-TIL therapies.^54^

The expansion of TILs from multiregional biopsy samples, from distinct cancerous lesions within the same patient or from different regions within the same tumour, could give a more accurate snapshot of intratumour heterogeneity at a single time-point (Fig. 1b) and, therefore, enable the more successful design of TIL therapies targeting a substantial proportion of tumour cells in any given cancer.^55^ Neoantigen-specific CD8^+^ and CD4^+^ lymphocytes have also been detected in peripheral blood,^56–58^ which could overcome the problem of limited specimen availability in certain tumours. However, the low levels of these neoantigen-specific lymphocytes from peripheral blood and their discordant TCR repertoire compared with TILs collected from the same patients could represent a challenge for their suitability for ACT.^59^

#### Clonal mutations and neoantigen quality

A 2020 publication looking at the timescales of tumour development showed that ‘first driver’ events seemingly occurred up to decades before diagnosis, demonstrating how cancer genomes are shaped by a near-lifelong process of somatic evolution.^60^ The evolution of most cancers usually involves common early mutations in cancer driver genes, followed by diverse trajectories generated by individually rare driver mutations, and by copy number alterations.^60^ Intriguingly, for some cancers, genomic sequencing is unable to identify driver mutations.^49^ A notable advantage of neoantigen-driven immunotherapy is that the issue of whether or not a mutation in a cancer gene occurs is irrelevant; what is important is that the mutation is clonal—that is, it is shared by all cancer cells—which increases the number of therapeutic targets in a way that is not feasible using traditional small-molecule targeted therapies. Predicting neoantigens that are shared amongst a substantial proportion of the target cell population is vital for the success of ACT, irrespective of which gene a mutation resides in. Mutations that occur early are important to consider for immunotherapy as they would be clonal and are, therefore, at the trunk of a tumour’s phylogenetic tree^61–63^ (Fig. 1b) and might consequently generate a more effective anti-cancer T-cell response compared with later mutations (subclonal) that are limited to subpopulations of cancer cells.^64–66^ The identification of clonal mutations relies on the accurate computation of the prevalence of mutations in a tumour.^67^ This is not a trivial task and is often confounded by normal cell contamination, substantial heterogeneity and copy number alterations.^68^ However, advances in sequencing and computational technologies, particularly in the field of linked-read methods, have enabled improved estimation of the prevalence of mutations by incorporating phase information. This allows the alleles identification on maternal and paternal chromosomes which is important for understanding gene expression patterns in tumours.^69,70^ Whether subclonal neoantigens developing in a rapidly evolving tumour or in response to treatment pressure^71^ can actively distract the immune response from effectively targeting clonal neoantigens is unclear. Thus, therapeutic efforts might need to be oriented toward the targeting of multiple clonal neoantigens to optimise disease control and minimise the potential for immune escape.

#### Predicting neoantigens beyond somatic mutations

Another key challenge is that only a few predicted neoantigens encoded by somatic non-synonymous mutations are actually immunogenic.^55,72–74^ Therefore, expanding the search for immunogenic antigens to include different categories of genomic alterations beyond non-synonymous mutations is essential. Single-nucleotide variants (SNVs), insertions and deletions (indels) causing frameshifts, chromosome alterations and splice variants could all potentially generate neoantigens (Fig. 1c).^75^ Data from cancer genomics studies also shed insights into the timing of acquisition of structural variants and^76,77^ copy number gains^78–80^ and the incidence and timing of punctuated events (such as chromothripsis, chromoplexy, kataegis) in cancer evolution.^49,81^ Owing to their high prevalence in cancer genomes and their near absence in healthy tissues, complex chromosomal rearrangements represent an exciting potential target for the identification of neoantigens. Frameshifts resulting from indels, if they evade nonsense-mediated decay, have been shown to generate approximately three times as many high-affinity neoantigens as non-synonymous SNVs.^82,83^ Interestingly, mitochondrial-localised peptides were shown to induce a more robust immune response than cytoplasmic peptides, owing to their increased stability, indicating that the subcellular localisation of peptides might be important in determining their immunogenicity, which could potentially be exploited during the identification of predicted neoantigens.^84^

As shown in Fig. 1a, the neoantigens predicted from genome sequencing data (whole-genome and exome sequencing) are usually filtered by gene expression (RNA-seq) to assess the expression (yes or no) of that given gene. However, single-cell transcriptomic sequencing (scRNA-seq) data from ovarian and lung cancer documented the presence of non-genetic heterogeneity amongst cancer cells, with evidence of remarkable plasticity and capability of transitioning from one cell state to another.^85–88^ This phenomenon might be relevant for the selection of cancer neoantigens. For example, knowing the predominant cellular state of a tumour (e.g., stemness programme^88^) would make it possible to prioritise neoantigens that are encoded by genes that are expressed in that particular cell state. Not surprisingly, promoter hypermethylation has been shown to affect the expression of ~23% of the predicted neoantigens studied in a large cohort of lung cancer,^89^ highlighting the occurrence of transcriptional repression mechanisms in cancer and their potential significance in neoantigen identification.^90^

#### Improving the prediction of neoantigen immunogenicity

Once potential neoepitope-generating mutations have been identified, T cells are assessed for their reactivity against tandem minigenes (TMGs) or peptides containing the potential neoantigens (Fig. 1a). Challenges in determining the anti-tumour reactivity of neoantigen-specific T-cells have been reviewed elsewhere^47^ but, in addition to technological limitations, it is important to note that not every specific neoepitope gives an immune response.^91^ An inherent bias in this pipeline is that we can only test the immunogenicity of the neoepitope-generating mutations that we are able to predict bioinformatically. In addition, neoantigen overlap predictions of the top 100 ranked peptide-bound MHCs from the same tumour samples between different teams of a global community has been shown to be low (less than 20%) in the majority of the cases. This lack of consensus might be partially driven by differences in epitope filtering and/or ranking between the different teams and suggest that efforts to harmonize neoantigen-prediction will be necessary for future clinical cross-comparison of neoantigen-based TIL trials between different centres.^92^ Moreover, despite mutations that poorly bind to HLA-II being positively selected for during tumorigenesis, thus emphasising the importance of CD4^+^ T cells in anti-tumour immunity,^93^ the computational prediction and analysis of HLA-II remains an ongoing challenge owing to the highly polymorphic nature of the HLA-II and poorly characterised endosomal HLA-II peptide processing, which limits the development of HLA-II peptide processing algorithms.^45,94,95^ Furthermore, despite the core binding motif of both HLA molecules comprising peptides of approximately nine amino acids, HLA-II-restricted ones have a wider length range (11–20 amino acids) compared with HLA-I-restricted ones (8–11 amino acids)^96^ which can make bioinformatics prediction task challenging.

The use of mass spectrometry for the direct discovery of tumour-specific HLA peptides (immunopeptidomics) holds promise for defining targets for immunotherapy (Table 2). Promises and challenges of this approach have been reviewed elsewhere.^97,98^ The use of data-driven machine-learning approaches to leverage information from established sets of HLA-I and, notably, HLA-II ligandomic data has provided new hope for improving the ability to predict a broader range of neoantigens, including those derived from post-translational modification as well as from the cancer-specific translation of products arising as a consequence of alternative splicing and intron retention, RNA editing, novel open reading frames and endogenous retrovirus elements^99–105^ (Fig. 1c). The need to incorporate these ‘unconventional’ tumour antigens into current approaches for predicting neoantigens has been reviewed.^38^ The increase in available data on peptide immunogenicity and TCR binding prediction along with machine-based learning extrapolation could have the power to improve the quality of neoantigen identification^106^ by incorporating structural and associated physical principles into approaches for evaluating immunogenicity^107^ and to potentially identify immunogenic hotspots for directed neoantigen targeting.^108,109^

**Table 2:** Glossary of terminology.

Neoantigen in silico peptide prediction and prioritisation	The indirect identification of candidate neoantigen-generating peptides derived from genome sequencing uses algorithms to identify those peptides that are more likely to be presented on HLA-I based on biochemical and biophysical properties. Algorithms for HLA-I-restricted peptides are less accurate for less frequent HLA-I clonotypes but overall more reliable than HLA-II predictors. Their performance depends on the accuracy of the used algorithms, which have shown low concordance in large studies
Neoepitopes and neoantigens	Neoepitopes indicate amino acid sequences that are presented by MHC molecules on a cell surface. Neoantigens refer to epitopes presented by a cell that contains sufficient MHC-peptide expression and induces a T-cell response
LC-MS/MS-based immunopeptidomics	Unbiased, direct identification of naturally presented HLA-bound peptides by liquid chromatography mass spectrometry. It also allows the identification of post-translationally modified peptides. Technical limitations still hinder it as it requires a discrete amount of tumour tissue and could be biased towards detecting the more abundant peptides. Also, it relies on the HLA expression of tumour cells
Techniques for identifying the immunogenicity of the predicted neoantigens	The experimental identification of which candidate neoantigens can generate an anti-tumour T cell response. Traditional methods involve the direct expression of putative neoantigens (with minigenes or peptides) within HLA-genotype matched antigen-presenting cells. These cells are then incubated with tumours to identify reactive T-cell populations by interferon (IFN)-γ enzyme-linked immune absorbent spot (ELISPOT), intracellular cytokine staining or using multi-colour-labelled major histocompatibility complex (MHC) tetramers for multiplex flow cytometry
MHC tetramers	Complexes of four MHC molecules associated with a specifically predicted neopeptide and bound to a fluorochrome
Tandem minigenes	A string of minigenes encoding the mutated amino acid flanked by 12 amino acids on their N- and C-termini
T-cell receptor (TCR) repertoire	This refers to all of the unique TCR genetic rearrangements within the adaptive immune system at a given time point. Evenness, richness, and diversity (typically used to describe ecological communities and their interactions with their environment) describe the TCR profile. Clonal evenness refers to the distribution of TCR to describe clonal expansion; clonal richness refers to measuring the number of clones with unique TCRs. Clonal diversity entails the distribution of TCR, taking into account both evenness and richness
Techniques for identifying the TCR repertoire	Bulk next-generation sequencing allows the high-throughput sequencing of α and β chains of TCR but is limited by the inability to pair α and β sequencing. Single-cell technologies can provide sequence information on paired α and β chains of individual cells and their association with the same cells’ gene-expression profile

In conclusion, despite being reliable and extremely promising, personalised immunotherapy that targets unique mutations faces many challenges. Apart from the technical and logistical hurdles for this highly personalised approach, which will be discussed below, a better understanding of cancer trajectories from preneoplastic lesions to invasive cancer and of the simultaneous pressure of the immune system (immunoediting) is warranted to improve the identification of neoantigens.^65^

## Cancer–immune system interactions

### Cancer immunoediting and immunoevasion

Scientific advances over the past decades have demonstrated that the immune system can paradoxically both constrain and promote tumour development and progression. This process is referred to as cancer immunoediting and, in its most complex form, involves three phases—elimination, equilibrium and escape—that ultimately result in the advent of immune-resistant variants.^110,111^ The constant pressure from the adaptive immune system coupled with the genetic instability of tumour cells can select for tumour subclones with reduced immunogenicity that can evade immune recognition and destruction.^112–114^ The immunological control of tumour progression and sculpting of tumour genomes has been shown in several solid cancers, with tumour regions that harbour the highest levels of immune infiltrates exhibiting the lowest cancer cell clonal diversity, which is likely to be a reflection of neoantigen depletion and loss of heterozygosity (LOH) at the HLA loci.^115,116^ The timing of initiation of immunological sculpting is an important question in cancer biology. The number of expanded TCRs found ubiquitously across all tumour samples in early-stage lung cancer or paired metastatic breast and ovarian cancer implies that some level of immune surveillance directed against clonal neoantigens can be initiated early and maintained through all levels of cancer development, including metastatic progression.^115,117,118^ However, as is evident through the lack of cancer cell elimination, immune escape mechanisms capable of preventing T-cell mediated death might be an extremely early event in cancer evolution.

#### Potential immune escape mechanisms

**Fig. 2:**
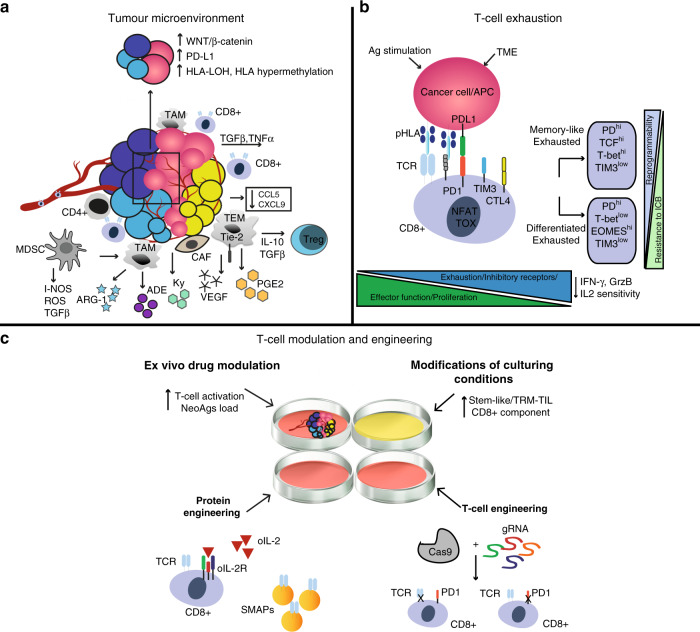
T cell conditioning to overcome the immunosuppressive tumour microenvironment. **a** Immuno-evasion mechanisms in the tumour microenvironment. A representative example of an ‘excluded’ (cold) T-cell tumour is shown. Some of the most studied immune cells along with their ligand–receptor and secreted growth factors (chemokines and cytokines) known to promote immunoevasion are shown. In the black box, examples are given of cancer genetic alterations linked to an immuno-evasive tumour microenvironment (ADE adenosine, ARG1 Arginase 1, CAFs cancer-associated fibroblasts, DC dendritic cell, IL interleukin, iNOS inducible nitric oxide synthase, KYN: kynurenine, MDSC myeloid-derived suppressor cells, NO nitric oxide, PGE2 prostaglandin E2, ROS reactive oxygen species TAMs tumour-associated macrophages, TGF-β transforming growth factor-β, Treg regulatory T cell, VEGF vascular endothelial growth factor). In the black box are highlighted genetic mechanisms linked to a cold TME. **b** T cell exhaustion in solid tumours. A representative image of transitions from an effector (T_eff_) to an exhausted (T_ex_) T cell is shown. Chronic antigen exposure and the TME pressure promote the activity of transcription factors (such as NFAT, TOX), which increases the expression of exhaustion-associated molecules such as PD1, LAG3, and TIM3, and the downregulation of effector cytokines such as IFNγ, GrzB and IL-2 sensitivity. GrzB Granzyme B, IL2 interleukin-2, TME tumour microenvironment, TRM tissue-resident memory. **c** Potential interventions to increase TIL efficacy during the expansion of T cells for ACT. The tumour microenvironment (TME) can be modulated ex vivo with different drugs (such as epigenetics, immunometabolic drugs) or interleukins (e.g. IL-2, IL-15) to boost the growth and activation of tumour-infiltrating lymphocytes, to increase the number of neoantigens (epigenetic drugs) or to preferentially expand TIL-specific subtypes such as tissue resident memory T cells (TRM-TILs). CRISPR–Cas9 ribonuclear protein complexes loaded with single-guide RNAs can be electroporated into TILs or normal T cells for gene editing. Shown here is an example of deletion of TCRs (off-the-shelf T cells) and of inhibiting immune checkpoint receptors such as programmed death 1 (PD-1) in T cells. Protein engineering can be used for the creation of orthogonal IL-2–IL-2 receptor pairs, which consist of a mutant orthogonal IL-2 cytokine (oIL-2) and mutant IL-2 receptor (oIL-2R) that interact specifically with each other but do not interact with their wild-type counterparts. SMAPs (supramolecular attack particles) can be produced in vitro and grafted to relevant specific TCRs for adoptive transfer.

The immunogenicity of neoantigens has been challenged. A study using data from The Cancer Genome Atlas (TCGA) showed that neoantigen depletion, detected using HLA affinity predictions, is weak or absent in the untreated cancer genome overall.^119^ This observation is consistent with the fact that the vast majority of non-synonymous mutations across 29 cancer types are not subject to selection and that only a minority of mutations (∼5%) were positively selected when evaluating the selection pressures exerted on mutant somatic cancer alleles (nonsense versus missense mutations).^120^ Similarly, few differences in the immunoediting of clonal neoantigens were seen in a large cohort of lung cancer patients.^89^ Therefore, either only very few predicted driver neoantigens are immunogenic, or driver mutations possess the ability to evolve efficient early immune evasion mechanisms. This could potentially be linked to individual variation at the HLA locus,^121^ LOH in the HLA region or by inducing other mechanisms such as amplification of the immune checkpoint molecule programmed death ligand-1,^119,122,123^ as was demonstrated by comparing preinvasive lesions of the lung that were immune-competent (and therefore regressed) with those that progressed.^124^ Additionally, hypermethylation of the HLA region was also commonly observed^124^ (Fig. 2a).

Interestingly, several studies have demonstrated the widespread appearance of cancer mutations in healthy tissues, including the oesophagus, skin, liver, lung, endometrium and colon.^125–130^ The concept of normal cell mutagenesis has revolutionised our understanding of cancer development, as driver mutations are not only limited to carcinogenesis but are also common events throughout life, occurring as early as embryogenesis.^131^ The presence of these widespread mutations in healthy tissue might also influence our understanding of cancer immunotherapy and cell-therapy development, as they could provide insight into those potential neoepitopes against which the immune system has been already tolerized (e.g. neoepitopes generated by mutations in cancer genes early in life). This might guide future prioritization of neoepitopes for neoantigen-based therapies.

Another explanation for the lack of negative selection of mutated cells is that the pervasive presence of driver mutations in healthy tissue starting even at the embryonic level is tolerated by the immune system until other insults, such as the potential invasion of the basal epithelial layers or metabolic changes, occur.^120^ In addition, some cancer-associated driver mutations have been reported to attenuate immune responses. For example, mutations in the genes *KRAS* and *BRAF* or other mutational activations in components of the mitogen-activated protein kinase (MAPK) pathway can decrease the transcription of HLA class I molecules as well as the expression of other genes encoding molecules that are essential for peptide loading.^132–135^ Whether and how these driver mutations in healthy tissues are surveyed, selected and removed by the immune system is a crucial biological question and could significantly enhance our understanding of cancer–immune interactions and the immunogenic properties of a given mutation.

Increasing our understanding will entail re-focusing on the importance of tissue-specific immunity/‘structural immunity’^136^ and how each organ’s immune system can affect cancer development and treatment.^137,138^ In the same way that comprehensive DNA profiling of tumours has revealed the genetics of cancers and the significant variation amongst tumours and individual patients,^49,60,81,139,140^ knowing the difference in the immunological composition and peripheral fitness selection of T cells^141^ between different organs might provide insights into the role of the early and late events of immunosurveillance in cancer development in a given tumour microenvironment (TME).^142,143^

### The tumour microenvironment and TILs

T-cell infiltration into the TME has been extensively demonstrated to be clinically relevant, with the quantity, quality and location of the immune infiltrate (known as the ‘immune contexture’) of cytotoxic and memory T cells within the solid tumour accurately predicting clinical outcome^144–147^ as well as a positive response to ICI therapy.^148,149^ The density of the T-cell infiltrate has been linked to cancer genomic characteristics, with tumours that are genetically more heterogeneous showing less immune infiltration.^150,151^ Analysis of synchronous metastases in patients with melanoma has revealed not only genetic heterogeneity but also immune-infiltration heterogeneity in terms of different immune cell types and T-cell clonality between different sites in the same patient.^152^

#### T-cell infiltration, activation and exhaustion

Relatively little is known about the cancer-intrinsic mechanisms, alterations and oncogenic signalling programmes that underlie TME heterogeneity.^153^ The dysregulation of specific pathways, such as WNT–β-catenin signalling, and tumoral amplification of genes that encode proteins associated with immunosuppression, such as PD-L1, the arachidonate lipoxygenases, and indoleamine-2,3-dioxygenase-1 (IDO-1) and IDO-2, have been linked with low T-cell infiltration and lower cytolytic activity.^154–156^ The association between WNT–β-catenin signalling and TME modulation is further supported by the observation that immune-excluded tumours are enriched for mutations in negative regulators of the WNT–β-catenin pathway in treatment-naive high-grade serous ovarian cancer^157^ (Fig. 2a).

Several biochemical pathways and cell types in the TME have been reported to lead to a decrease in T-cell infiltration (T-cell homing) and activation, as well as increased T-cell exhaustion, thereby increasing immunoevasion in different tumour types^158–161^ (Fig. 2a). It appears evident that an understanding of the biology of how the TME shapes the pattern and levels of immune-cell infiltration and exhaustion will be a key factor for the success of ACT.^162^ This understanding could provide the biological knowledge to pharmacologically modulate the key pathways of immunoevasion prior to or during the administration of ACT to the patients. Furthermore, drugs could be administered to tumour fragments ex vivo during the manufacturing of the cell product to increase TIL expansion and activation. In addition, the cell product could be engineered to enhance persistence and activation in vivo.^163^ These aspects will be discussed in further detail below.

#### Therapeutic approaches to increase T-cell infiltration

The ability of the TME to show a myriad of pathway redundancies and develop metabolic adaptations precludes, from a clinical point of view, multiple simultaneous targeting to avoid patient toxicity. However, the importance of establishing inflammation in the TME and thus overcoming immune exclusion and increasing T-cell homing in solid tumours (turning the tumours ‘hot’) has stimulated research into the discovery of new therapeutic options.^164,165^ Such an approach will be crucial for ACT success as the presence of TILs (indicative of an inflamed tumour) is a prerequisite for TIL expansion. Radiotherapy, which is capable of inducing immunogenic cell death by exposing tumour-associated antigens/neoantigens that can be recognised by antigen-presenting cells (APCs)^166^ and then presented to CD8^+^ T cells, is currently being tested in clinical trials in combination with ICB.^167^

The combination of ICB and ACT has been shown to be feasible and safe in patients with ovarian cancer.^168^ Another interesting strategy to render tumours ‘hot’ is the use of oncolytic viruses—native or genetically modified viruses that selectively infect and replicate within tumour cells, eventually leading to tumour cell lysis.^169^ Direct injection into cancer cells of the oncolytic virotherapy agent talimogene laherparepvec, which is also engineered to produce granulocyte/macrophage colony-stimulating factor (GM-CSF) to induce an immune response, increased T-cell infiltration and the response to ICIs in melanoma patients.^170^ Similarly, administration of a dendritic cell (DC)-based vaccine has been shown to increase T-cell infiltration and induce T-cell responses to autologous tumour antigens in ovarian cancer whole cell lysate.^166^ These therapeutic options could constitute a priming step to stimulate an immune response and thus create the basis for T-cell interrogation of neoantigens for subsequent TIL therapy, particularly in those patients whose tumours have a low number of TILs.^171^

These therapies (virotherapy, low-dose radiotherapy) can also induce a systemic increase of chemokines (such as CCL2 and CCL5) known to promote T-cell homing and T-cell infiltration^172–174^ (Fig. 2b). Indeed, a 2019 publication reported that the cooperation of chemokines such as chemokine ligand 5 (CCL5), which is constitutively expressed by tumour cells, with IFN-γ-inducible chemokines such as chemokine ligand 9 (CXCL9), plays a key and universal role in the orchestration of T-cell responses in tumours and facilitates the establishment of the T-cell-inflamed phenotype.^175^ The loss of tumour-intrinsic chemokines (such as CCL5, for example) that support T-cell recruitment is a common mechanism of immunoevasion. These results could open opportunities for the manipulation, using genome-engineering techniques, of these T-cell homing ligands in expanding TIL populations to enhance T-cell engraftment.

#### The preferential expansion of TIL subtypes

The intrinsic capacity of intratumoural T cells to recognise adjacent tumour tissue can be rare and variable (∼10% in the case of melanoma, ovarian and colorectal cancer CD8^+^ TILs^41,176^); the majority of TILs are bystander T cells.^177^ Thus, strategies to enrich a predefined neoantigen-specific subpopulation or tumour-specific cells would increase the chances of obtaining a final TIL product with adequate tumour reactivity as well as persistence in vivo.^178,179^ The preferential expansion, via cytokine modulation or using bioengineering materials, of specific T-cell subtypes, such as the tissue-resident memory (TRM) T cells, is an interesting approach.^180,181^ TRM T cells are particularly attractive for TIL-ACT as they are associated with better survival outcomes in many solid tumours and have an inherent capacity to home, owing to the expression of specific integrins on their cell surface.^182–186^ Moreover, the CD8^+^PD1^+^CD103^+^ TRM subpopulation represents the predominant proportion of TILs with expanded intratumoural ubiquitous TCRs (thus recognising clonal neoantigens), and these TCRs been demonstrated to be expressed on tumour-reactive T cells in lung cancer patients.^187^

As mentioned, the success of ACT depends not only on the homing capability of the cell product, but also on additional specific properties such as the differentiation state and ability to persist in vivo, along with the capacity to exert effector functions against cancer cells in the host (Fig. 2b). Therefore, a better understanding of the physiological mechanism that couples cell expansion and differentiation in T cells could improve the efficacy of ACT.^188^ An increased understanding might facilitate in vitro strategies to increase the percentage of tumour-reactive stem-like TILs, for example, which have been shown to be capable of self-renewal, expansion, persistence and a superior anti-tumour response in patients with melanoma.^189^

#### Maintaining T-cell activity/responses

The persistence of high amounts of antigen and the immunosuppressive nature of the TME push the majority of cancer-specific T cells towards an exhausted phenotype^176,177,190^ (Fig. 2c). As these cancer-specific T cells become less responsive to interleukin-2 (IL-2), which mediates T-cell expansion, they might become diluted by bystander T cells during the process of expansion in TIL-ACT, with an overall loss of TCR repertoire.^15,191,192^ However, despite their functional impairment, it is widely appreciated that exhausted T cells are often tumour-specific and can still retain some control over tumour growth, as shown by the great clinical effect of blocking programmed death protein 1 (PD-1) or programmed cell death ligand 1 (PD-L1) axis in solid tumours.^193,194^ Exhausted T cells express increased levels of inhibitory immune checkpoint molecules, such as PD-1, CTLA-4, LAG3 and TIM3,^195^ and decreased levels of the adhesion/costimulatory molecule CD2, both of which might attenuate anti-tumour T-cell responses in tumours.^196^ However, some subsets of exhausted T cells that express the transcription factor TCF1, also known as precursor exhausted T cells,^197^ display self-renewing capacity and are essential for the long-term maintenance of persistent T-cell responses in different solid tumours.^198–200^ The enrichment of this subpopulation during TIL expansion could therefore enhance the efficacy of ACT therapies.^189,201^

Future efforts to rapidly sort tumour-reactive cells (e.g. those expressing cell co-stimulatory molecules such as PD-1, 4-1BB or OX40)^59,191,202^ or metabolically ‘fit’ T cells^203^ might reduce the loss of efficacy seen in response to high-dose IL-2 culture conditions and improve the persistence of the T-cell product in vivo. Future technological advances that can integrate sensitivity into high-throughput approaches are warranted for the selection of a TIL product with better anticancer characteristics.^48,204,205^

So far, T cells in the earliest stages of differentiation (naive or central memory) have shown the highest efficacy and persistence in TIL-ACT regimens, as progressive terminal T-cell differentiation or exhaustion causes loss of anti-tumour power through impairments in TCR signalling and/or via reductions in either cytolytic activities or adhesion.^206,207^ However, although the isolation from patients’ blood of less-differentiated T-cell subsets can be an effective strategy for generating superior TCR or CAR-engineered T-cell products, it is more challenging when using TILs, which are often found in a state of senescence and functional exhaustion.^208,209^ Moreover, early states of T-cell differentiation might also coincide with a decreased expression of tissue homing molecules and trafficking potential, and a less preferential expansion of TRM T cells.^210^ Thus, preventing this kind of cellular fatigue (exhaustion), alongside manipulation of other molecular pathways, will help to unleash the potential of TIL-ACT for the treatment of solid tumours.^211^

#### Ex-vivo modulation of the TME

As mentioned above, the enrichment of TILs with better functional activity could potentially be obtained during the ex vivo expansion stage of the process. Notably, the TME was also able to be manipulated ex vivo by the direct application to cultured fragments of breast and ovarian cancer of the agonistic anti-CD137 antibody, which increased the rate of TRM-TIL outgrowth; and ICB has been shown to increase the polyclonal expansion of infiltrating CD8^+^ TILs and activation levels of the final T-cell product.^212,213^ Furthermore, the addition to expanding TILs of synthetic peptide pools of all predicted HLA-class I neoantigens improved conventional methods of TIL generation by enriching for neoantigen-specific CD8^+^ TILs.^59^

The use of epigenetic therapies in this ex vivo setting could increase the expression of transcriptionally repressed neoantigens and CTA,^214^ which could potentially enhance the recognition of cancer cells by adaptive immune cells.^215,216^ Similarly, epigenetic therapies could reverse the T-cell chromatin conformation and DNA methylation that are linked to decreased chemokine production, T-cell differentiation and exhaustion.^217–219^ Potential strategies to reprogramme the T-cell metabolic state ex vivo to improve T-cell phenotypes also exist.^220,221^ Treating expanding T cells with increased levels of extracellular potassium and acetate resulted in the generation of T cells with retained stemness, evidenced by self-renewal and multipotency.^222^ Other strategies have explored the inhibition of T-cell exhaustion by reducing mitochondrial oxidative stress,^223,224^ the reversal of T-cell senescence by inhibiting sestrin complexes.^225^

#### Immune-cell engineering

Notably, the time required to expand TILs could also be used as a ‘window of opportunity’ for immune-cell engineering with viral vectors^226–228^ or for gene editing with CRISPR–Cas9 technology.^229^ Loss-of-function studies in T cells have identified genes that, when deleted, can enhance T-cell responses,^230–234^ such as p38 MAPK (MAPK14).^235^ Many groups are also investigating whether candidate transcription factors, such as c-Jun,^236^ or synthetic cell receptors,^237,238^ such as the IL-2 receptor,^239^ can be overexpressed to reprogramme T cells, prevent exhaustion, or convert suppressive extracellular signals into activating signals. A first-in-human Phase 1 clinical trial of multiplex CRISPR–Cas9 gene editing in T cells from three patients with advanced, refractory cancer (two patients with myeloma and one with sarcoma) demonstrated that this approach is safe and feasible.^240^ T cells engineered using CRISPR–Cas9 editing to express a synthetic, cancer-specific TCR transgene and to lack expression of PD-1 persisted for longer than T cells retaining the expression of the endogenous TCR and PD-1. The gene editing and synthetic immunology areas are rapidly expanding, and universal approaches such as ‘off-the-shelf’ T cells (using T cells from healthy donors) generated through the editing of TCR could substantially advance the field and dramatically increase the number of patients eligible for cell therapies^241^ (Fig. 2d). Advances in protein engineering also hold promise. The expression of an orthogonal mutant IL-2 receptor and administration of its paired orthogonal mutant IL-2 ligand activates only the engineered T cells and not the IL-2 dependent CD4^+^Foxp3^+^ subpopulation of regulatory T (T_REG_) cells,^242,243^ which have been shown to decrease the therapeutic effect of ACT^244^ (Fig. 2d). Similarly, an engineered IL-2R agonist that preferentially reduces IL-2 binding to CD25 (low-affinity IL-2R, highly expressed in T_REG_ cells) over CD122/CD132 (high-affinity IL2R)^245^ was able to selectively expand intratumoural effector T cells over T_REG_ cells and synergise with anticancer vaccination.^246^

The 2020 description of the structure and composition of cytotoxic multiprotein complexes called supramolecular attack particles (SMAPs) released by CD8^+^ effector T cells (cytotoxic T lymphocytes; CTLs) and natural killer (NK) cells might open important avenues for ACT.^247^ SMAPs, which are composed of a core of cytotoxic proteins such as granzyme B and perforin and a shell of glycoproteins including thrombospondin-1, are a distinct type of extracellular particle of ~100 nm in diameter released by immune cells.^247,248^ Ideally, the innate specificity of these particles would be re-engineered towards an individual tumour and potentially HLA type, enabling the targeting of neoantigen-expressing tumour cells, potentially by grafting relevant TCRs onto their surfaces.

Several approaches exist that might, therefore, allow the development of optimised next-generation T cells that display better tumour selectivity, better tumour access capabilities, and increased activity in an immunosuppressive context. However, these approaches must meet rigorous quality control and regulatory qualification processes that account for the risk of off-target genome-editing modifications.

## Challenges in delivering personalised ATMPs

The promise of cell therapeutics for cancer treatment comes with new challenges in the form of reproducible manufacturing and in administering the product to thousands of patients,^249^ which requires the development of high-throughput and robust methodologies with high fidelity in a timely and GMP manner for therapeutic applications.^250^ Therefore, beyond the success of current clinical trials, commercial-scale cell therapeutics in cancer might not be available for many patients, simply due to a lack of capacity to deliver cell therapies. The associated logistical and economic factors—including physical space, production time, human resources, consumables and waste generation (and its environmental impact)—as well as other direct costs, are not trivial. All these factors must be integrated into the long-term view of a manufacturing blueprint.^251^

The application of cell therapy requires not only the manufacturing but also the distribution within a regulatory framework to ensure safety and efficacy. This framework also includes upstream events such as procurement of starting materials and the downstream storage and distribution of the product. The efficacy and stability of the final product are dependent on these processes, as they are on the rest of the key manufacturing process.^252^

### Commercial production of cell-therapy products

**Fig. 3:**
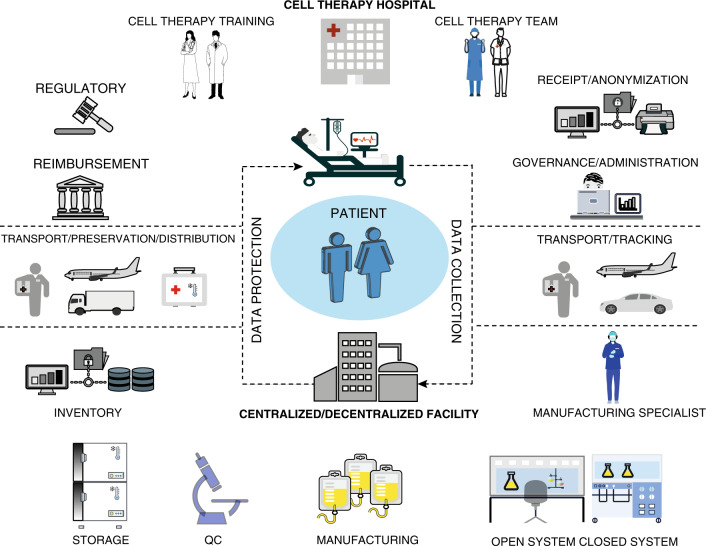
ATMP manufacturing chain and challenges. The chain starts with the cell-therapy team providing direct patient care. After apheresis or biopsy collection at the cell-therapy hospital, the material is anonymised, and the relevant setting is required to maintain a chain-of-custody tracking. The governance and administration team oversee the cell-therapy programme, the development and management of standard operating procedures, the outcomes of auditing processes as well as assess resource allocation and business planning. The biopsy sample is processed and transported to the manufacturing facility with a system to ensure that the integrity and chain-of-custody of the initial cellular material are maintained. At the manufacturing facility (centralised or decentralised), manufacturing specialists in GMP procedures with expertise in the standardisation of cell-therapy protocols are needed. Clean room requirements are determined by the use of open versus closed systems. A cell-product storage facility along with electronic database infrastructure for health record documentation and quality reporting preparation of manufactured products with a chain-of-custody are required. Transport of the cell product back to the hospital requires temperature and storage controls to maintain viability. In the hospital/organisation, a financial service dealing with single-case insurance agreements and institutional payer relations is required. A legal and compliance team overseeing the manufacturer contractual agreements, the interfaces with commercial manufacturers, as well as regulatory assessment for potential international trials, is also required. Similarly, staff education to provide proper clinical training and scientific and regulatory competencies (cell-therapy fellowships) are required.

Traditional manufacturing operations for human therapeutics are based on the ‘batch’ concept, in which the therapeutic is available ‘off the shelf’ for a large well-defined patient population. ATMPs instead target specific groups of patients or even individual patients using a sensitive live-cell therapeutic product. Efficient commercial production cannot therefore be achieved using the traditional processes implemented for biopharmaceuticals. Although many unit operations are similar, generating a cell-therapy product requires additional processing steps, such as cell selection, purification, formulation, preservation and distribution, all of which pose different technical challenges to those needed for the production of a molecular agent, especially in light of the number of modifications that cells are required to undergo (Fig. 3).^253^

The first consideration is that the recruitment of suitable patients to receive ACT is critical, particularly to TIL-therapy. The trial population should be carefully defined with a benefit–risk balance that should be positive at both a trial and an individual level. Patient recruitment for cell-based therapy treatments at an earlier stage, potentially before the use of ICB or other immunotherapies to ensure the presence of a polyclonal TCR repertoire in TILs or less senescent TILs,^199^ could facilitate the delivery of improved cellular products and minimise complicating co-morbidities that are associated with advancing metastatic cancer.^47^

### Manufacturing challenges in the ATMP pipeline

The issue of centralised versus small-scale/hospital manufacturing has been discussed elsewhere.^254–256^ However, in both cases, the production of patient-specific cell therapies presents unique challenges not seen in the manufacture of pharmaceuticals or biologicals.

Several academic hospitals worldwide have started to build their own cell-therapy programmes along with their own in-house manufacturing capacity.^256,257^ However, as only pharmaceutical companies have historically possessed the required manufacturing expertise to drive late-stage product development and manufacturing, government-funded initiatives to support the growth of cell-therapy organisations by bridging the gap between scientific research (academia) and full-scale commercialisation (industry) are warranted.

#### Analytics

As mentioned, rather than scaling-up to increase the batch size (and thus gain efficiency in the process), the processes supporting cell therapies need to be ‘scaled out’ to deliver a large number of individual batches.^258^ Therefore, with each dose representing a separate batch, the requirements for records, quality control (QC) testing, and quality assurance (QA) release must be repeated for every patient. The QC steps for cell therapy aim to ensure that the cell product is maintained bioburden-free and to verify the characteristics of the therapeutic product, such as identity, purity, activity, functionality, geno-/pheno-typic changes of the cells and the biocompatibility of the cell products, as well as other factors such as the culture medium. Developing new analytical technologies that will support in-process and the release of a high number of batches (by multiplexing QC tests) will be needed.^259^

#### Biological safety and standards

The provision of a sufficiently high degree of assurance that the environment does not contaminate each batch is of course necessary. A comprehensive analysis of previously unpublished industry-wide viral contamination information showed how viral infection of cell culture poses a real risk, particularly for cell therapy. In the context of ATMPs, safety relies almost exclusively on preventing contamination by using rigorous process controls.^260^ For these reasons, as cell therapies continue to advance quickly towards the clinic, the need to engineer robust, sustainable and cost-effective manufacturing processes becomes increasingly important.^261–263^

The biological activity of the raw materials used for cell expansion (e.g. cytokines) is of paramount importance as this can significantly alter the potency and safety profile of cell products and have significant implications on clinical outcomes. Indeed, as previously mentioned, various groups are testing different sources of materials and cytokine mixture to improve cell expansion and isolation. However, the range of approaches and assays to test T-cell efficacy against autologous tumours, the variation in processes and the various equipment used could hamper comparability and cross-validation of data.^259^ Consensus best practices and measurement assurance guidelines must thus be developed along with eventual standards.

#### Automation

Several closed automated systems have been developed to reduce the need for a higher class of cleanroom and labour-intensive processes that limit the supply, demand high production costs, and ultimately hinder investments. Functionally closed systems enable the appropriate degree of sterility and automation needed and allow the implementation and modulation of the product on a GMP level.^264^ The industrialisation of these operations on a small-scale level through robotics and novel types of equipment will provide, on a single platform, the necessary scalability, by parallel processing of multiple patient batches, for large clinical trials^263,265^ It will also allow the manufacturing of multiple personalised products within a small sized GMP facility.^256^ With the need to select a high number of specific cell subtypes for neoantigen-TIL therapy, future technological improvement of cell-sorting instruments at GMP grade is also required.

#### Qualified personnel and accredited centres

One of the main challenges for the success of a cell therapy programme will be the availability of qualified personnel sufficiently trained in the processes and operations of a GMP facility. A 2017 survey established there were approximately 500 roles in bioprocessing in the UK, while a similar 2019 survey demonstrated significant growth beyond the expected forecast, with current bioprocessing employment at >1,700 roles; this figure is forecast to reach over 3,800 by 2024 in the UK.^266^ Existing academic initiatives, as well as new bridges between academia and industry for training, need to be expanded to meet the current demand for GMP manufacturing specialists.^266,267^

Another critical point is the designation of cell-therapy accredited centres that have shared and agreed clinical and laboratory standards of excellence.^268^ Cell-therapy programmes require specialised professional multidisciplinary teams that are focussed on addressing the complex infrastructure and patient care needs inherent to such programmes.^269^ The incorporation of a structured audit as a requirement for accreditation is warranted due to the high standards required for successful cell-therapy programmes.^270^

#### Transport and storage

A key consideration is transport, which can have a significant impact on the product’s viability. The transport of newly harvested biological materials to a manufacturing centre close to the collection point simplifies the logistics and in parallel decreases the risks related to the deterioration of cellular product quality and integrity.^267,271^ In addition, for transportation to a central manufacturing facility, the issues for data inventory, packaging, and tracking become even more critical.

The need to store cellular material or conserve particular cellular attributes, often at several points during the manufacturing and transportation processes, necessitates the optimisation of cryopreservation of cell-therapy products, as these products are highly susceptible to temperature fluctuations. Suboptimal cryopreservation can lead to batch-to-batch variation, lowered cellular functionality and reduced cell yield.^272,273^ Further research and development, as well as the establishment of regulatory guidelines, will be required to ensure robust and reproducible approaches to the freezing, storage and thawing of the product.^274^

### Challenges for the widespread availability of ATMPs

The Food and Drug Administration projected that by 2025 it would be approving between 10 and 20 new cell and gene therapy products a year with more than 40 of these therapies potentially available on the market in the next five years. The incorporation of cell and gene therapy as a new therapeutic strategy, along with surgery, chemo and radiotherapy, will determine critical new challenges in the clinical practice. In fact, the complexity of these treatments is not limited only by manufacturing issues, but also by a new paradigm shift in cancer treatment, where the patients and hospital personal are part of the supply chain with all the training, ethical and legislative issues connect it.

#### Personnel, networks and communication

The transition from drug-based clinical trials to a growing number of cell therapy trials will also increase the need for skilled clinicians in the field of ATMPs, both from a scientific and regulatory point of view. Cellular therapy fellowships are starting to be advertised in the USA and will probably be implemented worldwide. These schemes should not be restricted to immuno-oncologists but, rather, should aim to create multidisciplinary teams comprising surgeons, interventional radiologists and other allied specialists dedicated to cell therapy programmes. A critical step in this direction would be to establish specialised treatment centres for ATMPs that enable networking activities between manufacturing units, specialised contractors, academic research, clinical centres, patients and caregivers. These networked clinical environments would also facilitate new business models, in which decision-making, cost, and risk of establishing efficacy, safety and quality are shared through an infrastructure that links data across cell-therapy professionals and stakeholders at all stages of development.^275^ These networked clinical environments will also provide an opportunity to address the evidence requirements for licensure and reimbursement, allowing stakeholder connectivity around post-authorisation commitments. The need to actively involve/engage patients/patient groups as key stakeholders in discussions around the development of ATMPs will be crucial. Support and information should be available for patients—and caregivers—undergoing treatment with ATMPs. Patients should be fully informed about the treatment and its effects through discussion with a healthcare professional and provision of patient resources.

#### Access to ATMPs

It should be acknowledged that access to ATMPs is likely to be a particular challenge for patients, healthcare professionals and national health systems, owing to their expected high costs. In particular, the cost of ACT using mutation-reactive T cells can be several times that of CAR-T cell-based immunotherapy.^47^ Not surprisingly, access to haematopoietic stem-cell transplantation is still associated with higher-income countries only.^276^ Although a discussion of the reimbursement aspect of ATMPs is outside the scope of this review, ensuring equitable access to cell therapies, from a financial and geographical point of view, and managing expectations and patient demands for access to therapeutics with high potential but as-yet-unproven interventions are important topics.^277^

#### Ethics and regulatory requirements

Professional communities should also promote the adoption of standards that meet the arising associated ethical considerations. An integrated ethical approach that aims for transparency and regulation of development processes, the support of independent judgement and the elimination of unregulated and uncontrolled grey areas of action are necessary to move cell therapy forward.^278^ Therefore, national and international societies should make readily available up-to-date information on accredited centres to minimise the risk of unproven and unethical interventions to patients.^279^

International co-ordination in the harmonisation of regulatory requirements is warranted because of potential future multicentre ATMP trials. In fact, specific regulations still vary by nation: for example, in the European Union and Switzerland, GMP requirements are expected for all stages of clinical trials (including Phase 1 trials where most novel ATMPs are located), but this is not the case in the USA.^253,280^ Regulations and standards must also evolve to reflect products that are personalised and sometimes administered in settings of urgent medical need.^281^ Ideally, these initiatives should be led by an umbrella of international societies to avoid duplication of effort.^282–284^

## Conclusions

The field of ACT is growing exponentially. However, despite the complete and durable responses seen in some patients with late-stage and treatment-refractory diseases, a subset of patients do not respond, might relapse, or do not have an adequate ACT strategy for their disease.^285^ New strategies and ideas are needed to enhance T-cell efficacy and reduce exhaustion, to circumvent TME immunosuppression and to optimise T-cell manufacturing. Future translational efforts might open the way for combinational or sequential therapies with ACT-TIL. Parallel processes are required to establish treatment centres, modernise regulatory and commercialisation approaches and to effectively deal with arising ethical considerations. The involvement of all stakeholders, including patient representatives, will be essential to ensure the successful translation of cell-therapy research to clinical implementation.

Here, we have reviewed the three pillars of ACT—cancer genomics, cancer–immune system interactions and cell therapy manufacturing—and discussed ongoing research efforts in these fields that will hopefully broaden the application of ACT to many cancer types. The integration of new genome-engineering or genome-editing technologies such as CRISPR into cell therapies might lead to significant progress.^286^

Issues of scale-up, automation and commercialisation that are unique to ACT need to be addressed to create the infrastructure required for the widespread availability of cell-therapeutics strategies. Finally, new models of academia–industry collaboration will be required. We argue/believe that overcoming the challenges facing ACT in such processes has the potential to improve clinical outcome for patients with a wide variety of cancer types.

## Data Availability

Not applicable.
